# Developmental plasticity, cell fate specification and morphogenesis in the early mouse embryo

**DOI:** 10.1098/rstb.2013.0538

**Published:** 2014-12-05

**Authors:** Ivan Bedzhov, Sarah J. L. Graham, Chuen Yan Leung, Magdalena Zernicka-Goetz

**Affiliations:** Department of Physiology, Development and Neuroscience, University of Cambridge, Downing Street, Cambridge CB2 3DY, UK

**Keywords:** embryo, morphogenesis, cell fate, differentiation, pluripotency

## Abstract

A critical point in mammalian development is when the early embryo implants into its mother's uterus. This event has historically been difficult to study due to the fact that it occurs within the maternal tissue and therefore is hidden from view. In this review, we discuss how the mouse embryo is prepared for implantation and the molecular mechanisms involved in directing and coordinating this crucial event. Prior to implantation, the cells of the embryo are specified as precursors of future embryonic and extra-embryonic lineages. These preimplantation cell fate decisions rely on a combination of factors including cell polarity, position and cell–cell signalling and are influenced by the heterogeneity between early embryo cells. At the point of implantation, signalling events between the embryo and mother, and between the embryonic and extraembryonic compartments of the embryo itself, orchestrate a total reorganization of the embryo, coupled with a burst of cell proliferation. New developments in embryo culture and imaging techniques have recently revealed the growth and morphogenesis of the embryo at the time of implantation, leading to a new model for the blastocyst to egg cylinder transition. In this model, pluripotent cells that will give rise to the fetus self-organize into a polarized three-dimensional rosette-like structure that initiates egg cylinder formation.

## 1. Heterogeneity guides preimplantation cell fate decisions

### (a) Regulative development of the preimplantation embryo

**Figure 1.**
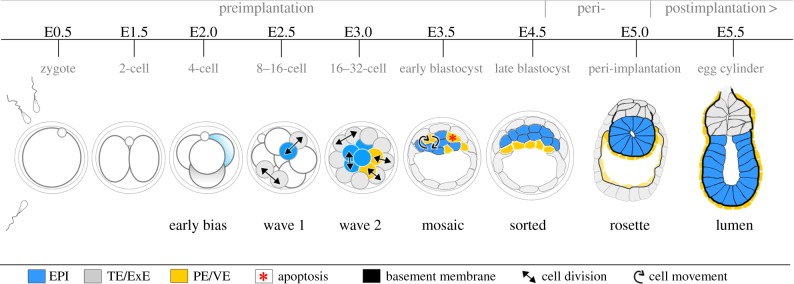
Overview of early mouse development. Embryonic and extraembryonic cells are specified in the preimplantation embryo by two cell fate decisions. In the first cell fate decision, waves of cell divisions create inside and outside cells. Outside cells give rise to extraembryonic trophectoderm (TE), while inside cells form the pluripotent inner cell mass (ICM). In the second cell fate decision, cells of the ICM are segregated into the extraembryonic PE and the pluripotent epiblast (EPI) that will later give rise to all tissues of the body. These fate decisions are influenced but not determined by heterogeneity between individual cells within the embryo that is established by the 4-cell stage (shown by different shading of cells). At E4.5, the embryo initiates implantation and over the next 24 h invades the maternal tissues, rapidly proliferates and transforms into an egg cylinder. This new form serves as a foundation for EPI patterning, laying down the body axis and establishment of the germ layers. ExE, extraembryonic ectoderm; PE, primitive endoderm; VE, visceral endoderm.

Mouse preimplantation development involves the sequential division of the fertilized egg into progressively smaller cells, or blastomeres, over the first four and a half days of life. This process results in the formation of a hollow ball of cells, the blastocyst, which is capable of implanting into the maternal uterine wall and comprises the necessary cell types to give rise to both embryonic and extraembryonic tissues in later development (figure 1). The blastocyst is organized into three distinct cell lineages by the time of implantation: the extraembryonic trophectoderm (TE) and primitive endoderm (PE), and the embryonic epiblast (EPI). The specification of these three cell types is achieved through two ‘cell fate decisions'. In the *first* cell fate decision, two major waves of asymmetric cell divisions at the 8- to 16- and 16- to 32-cell transitions and a minor wave at the 32- to 64-cell transition generate outside and inside cells that differ in their cellular properties, position within the embryo and their fate [1–3]. Outside cells will differentiate into TE, the precursor lineage of the placenta. Inside cells form the pluripotent inner cell mass (ICM) and will be further separated in the *second* cell fate decision into the differentiating PE that predominantly gives rise to the yolk sac, and the pluripotent EPI that is the precursor of the future fetus. The correct specification and organization of these different cell types is essential for development of the embryo beyond implantation, and how they are specified from a small cluster of seemingly identical cells is a fundamental question of mammalian developmental biology.

Understanding how cell fate is specified in the pre-implantation embryo has been complicated by the flexibility of early mammalian development. Early experiments manipulating the preimplantation mouse embryo demonstrated that its development is regulative, that is it can adapt and compensate for perturbations in the positions and numbers of cells. Removing blastomeres, rearranging them or making chimaeras of more than one embryo can all result in the formation of a blastocyst, indicating a flexibility in cell potential until the 32-cell stage [4–7]. This ability of cells in the embryo to modulate their fate in response to contextual changes led to the hypothesis that early development was driven by entirely random processes, with all cells equally able to contribute to any lineage [8]. However, this raises the question—if all cells are the same, how do they know what to do? The most obvious way in which cells can be different from each other is their position within the embryo, with outside cells developing into TE, surface ICM cells becoming PE and deep ICM cells becoming pluripotent EPI. Position can indeed alter cell fate [7,9–11] and this position model is attractive in its simplicity. However, recent discoveries indicate that cell position is not the only factor involved in controlling cell fate in the mouse embryo. For example, it was discovered that cell fate can be altered in the first cell fate decision by modifying the expression of specific genes, which in turn leads to a change in cell position [12]. The primary role of position in the second cell fate decision has also been challenged by the observation that the precursors of the PE and EPI are initially mixed within the ICM, before being sorted into their correct positions by active cell migration and selective apoptosis [13–15]. These findings demonstrated that position is not the only factor driving both the first and the second cell fate decision and suggested that rather than cells becoming different from each other in response to their positions, they are already biased towards certain fates before they reach distinct positions.

### (b) When do cells first become different from each other?

Recent advances in technologies that allow the tracking of individual cells throughout preimplantation development have provided insights into when these early differences arise. The first experiments that involved tracking cells labelled with *in vivo* markers suggested that blastomeres are different from each other already at the 2-cell stage. By non-invasively labelling blastomeres or marking the zona pellucida after the first cleavage division, it was demonstrated that both 2-cell stage blastomeres contribute to all blastocyst lineages, but they are biased towards contributing more to either an extraembryonic or an embryonic part of the embryo [16–18]. This view was challenged by reports that concluded the embryonic/extraembryonic axis of the blastocyst was determined by physical constraints of the embryo within the zona pellucida rather than any inherent differences between blastomeres [19,20]. More information on this subject was provided by the discovery that 4-cell stage blastomeres differ in their developmental potential depending both on whether they are daughters of the first or second-dividing 2-cell blastomere and on their division orientation with respect to the animal–vegetal axis of the egg. It was demonstrated that one blastomere that contains the most vegetal portion of the embryo is biased to give rise to extraembryonic lineages rather than embryonic ones [21,22]. The reduced developmental potential of this cell was found to relate to lower levels of histone H3 methylation on specific arginine residues, demonstrating for the first time that 4-cell stage blastomeres differ at an epigenetic level [23]. These results were disputed [19,24] but a recent genetic lineage tracing study using Rainbow mice confirms a significant lineage bias being initiated at the 4-cell stage [25]. In addition, several studies have identified heterogeneous expression or activity of specific genes in 4-cell stage blastomeres [26,27], providing further evidence that individual cells are different from each other already at this early developmental stage. Studies tracking the fate of individual cells have begun to uncover how these early differences might influence cell fate decisions.

### (c) Heterogeneity and cell fate

The first molecular difference to be identified between cells at the 4-cell stage was differences in the levels of arginine methylation of histone H3, specifically R26 and 17. This methylation was found to be highest in cells destined to contribute to the ICM and lowest in the cells that will give rise to the TE. In agreement with this, high H3 methylation levels correlates with increased expression of Nanog and Sox2 [23]. The cells biased towards contributing to extraembryonic lineages preferentially divide symmetrically and thereby avoid contributing to the ICM [28]. A clue to how division orientation could be influenced by the developmental history of cells came from the observation that at the 8-cell stage some cells express higher levels of the TE-specific transcription factor Cdx2 [29–31]. Tracing the history of these cells revealed that they are derived from the vegetal 4-cell blastomere known to be biased towards an extraembryonic fate [31]. Consistently, up- or downregulating Cdx2 expression level affects division orientation, most likely by influencing the apical–basal polarity of the cell [31]. In this way, the heterogeneity of cells in the embryo provides a mechanism by which blastomeres can be influenced when undertaking the first cell fate decision.

Heterogeneity between cells also influences the second cell fate decision. In the early embryonic day (E) 3.5 blastocyst, ICM cells express markers of the PE (Gata6) and EPI (Nanog) in a mosaic ‘salt and pepper’-like distribution, with this early gene expression pattern identifying the precursors of each lineage [13,15,29]. Without this coordinated expression of Gata6 and Nanog in the ICM, correct specification of the PE and EPI fails [13,32]. Whether this heterogeneity results from stochastic fluctuations in gene expression or whether ICM cells are different from each other due to their developmental history has only recently been possible to address. Several independent studies demonstrated that the fate of ICM cells is biased by the timing of cell internalization, with inside cells generated earlier more likely to contribute to EPI and those arriving inside later biased to form PE [2,33,34]. By generating the ICM in sequential waves of asymmetric divisions, the embryo has an in-built mechanism for creating a heterogeneous ICM cell population. This bias relates to the reciprocal expression of fibroblast growth factor (Fgf)4 ligand and Fgf receptor (Fgfr)2 in the precursors of the EPI and PE respectively, with cells internalized earlier upregulating Fgf4 and those internalized later expressing higher levels of Fgfr2 [33,35]. Single-cell RNA sequencing of isolated ICM cells confirmed differential expression of Fgf ligands and receptors to be a feature of the ICM prior to the ‘salt and pepper’ expression of Gata6 and Nanog and highlighted the importance of heterogeneity within the ICM for the establishment of two different cell fates [8]. The importance of this internalization-time bias depends on the ICM composition, having the greatest influence when both waves of asymmetric divisions contribute equally to the ICM, most likely due to the ratio of cells expressing Fgf4 and Fgfr2 in the embryo [33,35]. This context-dependent bias demonstrates how position, signalling and inherent heterogeneities all influence cell fate decisions. Heterogeneity therefore provides biases that guide, but do not determine, cell fate decisions thus allowing flexibility. Strikingly, without this heterogeneity, the developmental potential of embryos can be compromised, as seen in chimaeras generated from ‘like’ 4-cell stage blastomeres [21,36].

## 2. The molecular basis of preimplantation cell fate specification

### (a) Transcriptional identities of the trophectoderm and inner cell mass

Several transcription factors identified as important for ICM specification, such as Oct4 [37], Nanog [38] and Sox2 [39], are initially expressed in all cells of the morula, with their expression gradually becoming restricted to the ICM. By contrast, the transcription factors important for TE specification are restricted earlier, at the 8- to 16-cell transition. These markers include Cdx2 [40,41], Eomes [42] and Gata3 [43]. The TE and ICM identities are incompatible with each other and indeed Cdx2 and Oct4 reciprocally inhibit each other [41].

One of the important functions of the TE is the formation of the blastocyst cavity, the location of which determines the embryonic–abembryonic axis of the embryo. As the TE matures, it forms a polarized epithelium with intercellular junctions creating a permeability seal between the cells that allows for cavity expansion [44–47]. The initiation of cavity formation does not depend upon cell number but rather the formation of these junctions [48,49], and the cavity typically forms in regions where the outside cells are daughters of symmetric 16- to 32-cell divisions [28]. Cavitation is initiated around the 32-cell stage by diffusion of water across osmotic gradients and the transport of water through aquaporins on the apical and basolateral sides of TE cells [50,51]. The exact regulation of this process still remains unknown.

To understand cell fate specification, it is critical to determine how the reciprocity in the expression pattern between TE and ICM markers is first established. Activation of the TE fate programme in outside cells is regulated by the transcription factor Tead4, depletion of which leads to the earliest thus far reported embryonic lethal TE phenotype [52,53]. Although elimination of Tead4 does not affect either cell adhesion or polarization, expression of the TE transcription network is compromised and expression of Oct4 in outside cells is maintained [52,53]. Highly homologous family members Tead1 and Tead2 (but not Tead3) are also expressed during preimplantation stages, but do not appear to have a role before implantation [54,55], indicating that Tead4 is a non-redundant master regulator of the TE fate. This importance of Tead4 led to an expectation that its expression would be restricted to TE progenitors, however it was found to be expressed constitutively in not only outside but also inside cells of the blastocyst [53]. A further layer of regulation must therefore activate Tead4 selectively in TE progenitors. In the preimplantation embryo, Tead4 activity requires the two homologous transcriptional co-activators Yap and Taz, which are regulated by the core Hippo signalling pathway kinase Lats1/2 [56]. An active Hippo pathway prevents Yap and Taz from reaching the nucleus and, consequently, Tead4 activity is switched off and its target genes silenced. Yap and Taz are also expressed uniformly in the embryo but have distinct intracellular distribution: they are nuclear in outside cells, where Tead4 is active and the TE programme is activated, and cytoplasmic in inside cells, where Tead4 is inactive resulting in an ICM programme. Neither Yap nor Taz alone are essential for preimplantation development [57–59], but *Yap*^−/−^; *Taz*^−/−^ double mutants die before the 16- to 32-cell stage [56], resembling the *Tead4*^−/−^ phenotype. As expected *Lats1*^−/−^; *Lats2*^−/−^ double mutant embryos have increased nuclear Yap and express Cdx2 in inside cells [56]. The differential localization of Yap and Taz in inside and outside cells is therefore critical for the activation of specific transcriptional programmes in the first cell fate decision. The activation of the TE fate programme in outside cells is reinforced by a positive feedback loop in which the expression of Cdx2 inhibits the expression of pluripotency genes and positively regulates its own expression [41]. Inside cells are at least partially protected from the activation of this programme by the exclusion of Cdx2 mRNA from inside daughter cells of asymmetric cell divisions [60]. This asymmetric inheritance of Cdx2 transcripts has been shown to be dependent upon the apical localization of Cdx2 mRNA as the 8-cell stage blastomeres develop apical–basal polarity. Disruption of this mRNA localization leads to expression of Cdx2 in inside cells and a reduction in the number of pluripotent EPI cells [60], which compromises developmental potential [36]. Together, these findings highlight the importance of creating cells with different positional identities for the generation of separate embryonic and extraembryonic lineages. A key question remaining, however, is how positional information is connected to the differential localization of Hippo pathway components in inside and outside cells.

### (b) Polarity translates positional cues into molecular differences

An intrinsic difference between inside and outside cells is cell–cell contact. At the 8-cell stage, all cells are positionally equivalent. The embryo then undergoes compaction, in which the blastomeres adhere tightly to each other, and the cells become polarized along their apical–basal axis. When inside cells are generated, they lose polarization and encounter uniform cell–cell contacts. By contrast, outside cells remain polarized and have asymmetric cell–cell contacts, with an apical domain forming at the bare patch of membrane that is not in contact with neighbouring cells. Apical polarity factors such as Par3 and atypical protein kinase C (aPKC) accumulate at this apical domain and interference with components of the Par complex has been shown to affect both cell position and cell fate [12,61]. This discovery, combined with the observation that polarity and cell adhesion are not affected in *Tead4*^−/−^ embryos [53], indicates that the apical polarity complex is upstream of the TE fate programme. The basolateral domain, where cells are in direct contact with each other, contains adherens junctions (AJs) that mediate cell adhesion via E-cadherin. In E-cadherin mutants, cell adhesion is compromised and the apical domain extends over the entire cell [62]. Embryos lacking both maternal and zygotic E-cadherin have more Cdx2-positive cells, suggesting that when all cells of the embryo are polarized, the majority will take on a TE fate. Indeed, in these E-cadherin maternal/zygotic mutant embryos, cells with membrane-enriched aPKC show nuclear localization of Yap and express Cdx2, independent of their position within the embryo [62]. This implies that the establishment of apical polarity is dominant over cell position in cell fate. Similarly, the asymmetric cell–cell contact of isolated ICMs leads to polarization [62] and TE cells are regenerated [11,63], demonstrating that ICM cells retain plasticity and can change their fate in response to new positional information. Cell–cell contact can therefore be viewed as an inhibitor of apical domain formation and the internalization of cells by asymmetric divisions as a mechanism by which embryonic cells can be protected from differentiation cues. If part of the membrane loses cell–cell contact, as is the case in outside cells, an apical domain is established, leading to the activation of a dominant TE fate programme.

But what is the connection between the Hippo pathway and polarity? Recent studies have identified angiomotin (Amot) as a missing link. Amot and angiomotin-like 2 (Amotl2) are homologous membrane-associated Hippo pathway components that are differentially localized in inside and outside cells: in inside cells Amot is present throughout the membrane, whereas in outside cells its localization is apical [61,64]. It has been discovered that loss of Amot leads to nuclear localization of Yap and, consequently, expression of TE markers in the ICM [64]. This reduces embryo viability during peri-implantation development, particularly when both Amot and Amotl2 are depleted together, indicating that Amot, like Lats1/2, is required to suppress the TE programme in the ICM. Amot is an activator of Lats2 [65] and consequently can activate the Hippo pathway in the embryo [61]. However, Amot has also been shown to be able to sequester a Hippo pathway-insensitive Taz mutant, indicating that Amot can also inhibit Yap/Taz activity by physical tethering [66]. In agreement with this possibility, overexpression of Amot can compensate for the loss of Lats1/2 in the embryo [64]. Because endogenous levels of Amot do not seem able to suppress the TE programme in *Lats1*^−/−^; *Lats2*^−/−^ mutants [49], this Hippo-independent action of Amot is unlikely to be dominant. Interestingly, Amot itself can be phosphorylated by Lats2, which is required for the activation of the Hippo pathway [61] and could represent a positive feedback loop between Amot and Lats. As Amot has a polarized distribution in outside cells, it was hypothesized that apical polarity determinants may inhibit Amot, allowing for the TE cell fate. Indeed, PKCλ and Par6 are required to anchor Amot to the apical domain in outside cells [61], and in their absence Amot distributes uniformly across the membrane and Yap becomes phosphorylated. Amot is likely to be tethered to AJs via interaction with Nf2, a Hippo pathway component [67,68]. Although localization of Nf2 is not polarized in outside cells, loss of Nf2 phenocopies *Amot*^−/−^ and *Lats*^−/−^ with accumulation of Yap in the nucleus of ICM cells and expression of TE markers [69]. As exogenous Lats2 can rescue the loss of Nf2 phenotype, Nf2 seems to be upstream of Lats [69]. However, overexpression of either Nf2 or Amot does not suppress the TE programme in outside cells, unlike overexpression of Lats2 [64,69]. This indicates that high levels of Nf2 or Amot cannot overcome the inhibition posed by apical polarity factors and that this does not apply to Lats. Moreover, outside cells actively degrade Amot, which does not occur in inside cells [64]. Thus, multiple regulatory factors in outside cells are involved in regulating Cdx2 expression to establish the TE fate programme in the mouse embryo.

**Figure 2.**
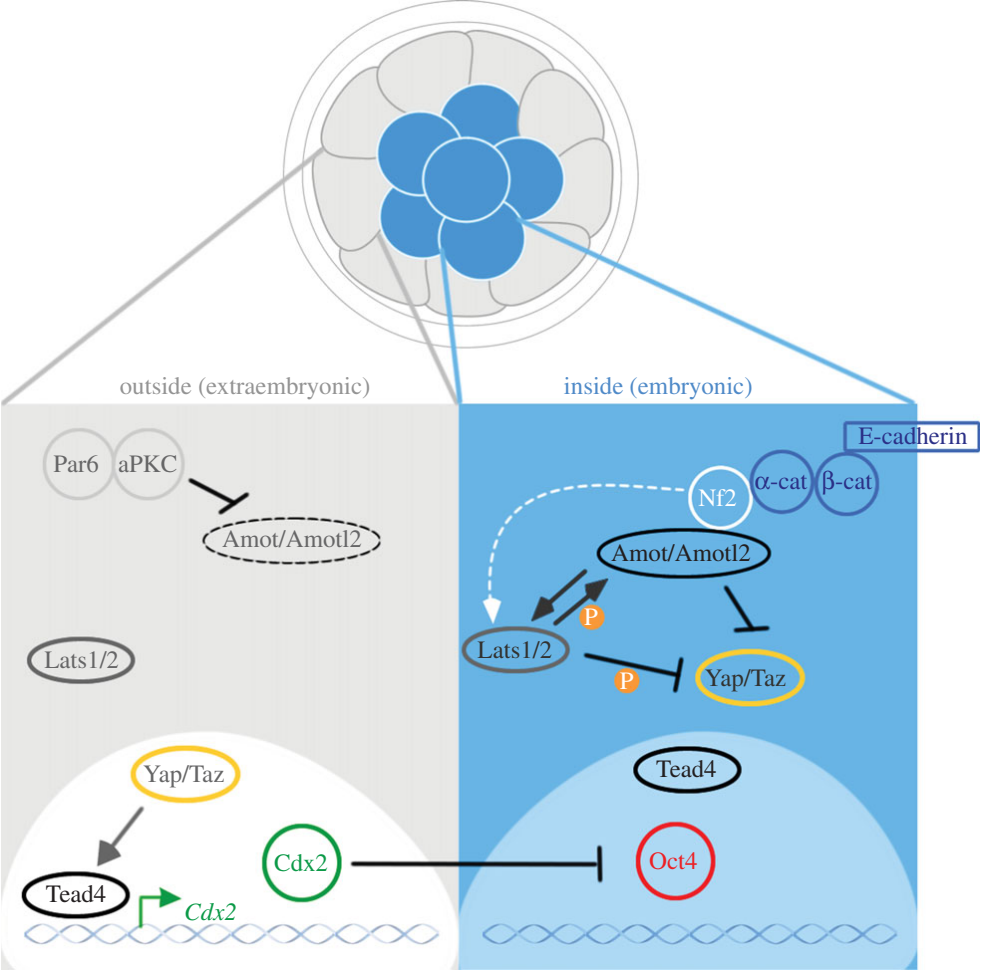
Specification of the TE and ICM. The compacted morula is the earliest point where blastomeres have differential spatial positioning. Blastomeres on the inside of the embryo encounter symmetric cell–cell contact and give rise to the pluripotent ICM. Outside cells have asymmetric cell–cell contact and form the extraembryonic TE. The asymmetry in cell–cell contact leads to accumulation of polarity proteins such as Par6 and aPKC at the apical domain. Par6 and aPKC activity inhibits activity of Amot and the Hippo pathway kinases Lats1/2. As a result, Yap and Taz are de-repressed, Tead4 transcriptional activity is switched on and Cdx2 expression and the TE cell fate programme are activated. In inside cells, symmetric cell–cell contact prevents establishment of an apical domain. Amot is active and is likely tethered to AJs through a putative Nf2–α-catenin–β-catenin–E-cadherin complex. Amot sequesters Yap/Taz to the cytoplasm as well as activating the Hippo pathway proteins Lats1/2. Lats1/2 can inhibit Yap/Taz activity via the canonical Hippo pathway and can also activate Amot in a positive feedback loop. In the absence of Yap/Taz in the nucleus, Tead4 activity is switched off, Oct4 expression is promoted and the default pluripotent programme is expressed. Therefore, positional and polarity cues in these two populations lead to changes in gene regulation and differentiation into their respective lineages.

These findings lead us to propose a model of how the TE and ICM are specified in a compact embryo containing outside and inside cells (figure 2). On the surface of the embryo, asymmetric cell–cell contact leads to the establishment of an apical domain, characterized by the Par3/Par6/aPKC complex. In the presence of apical polarity, the TE fate suppressors Amot and Lats are anchored to the apical domain and their activities are inhibited. Yap and Taz are allowed to enter the nucleus to activate Tead4 target genes, such as Cdx2, and consequently the TE fate programme is switched on. In the centre of the embryo, uniform cell–cell contact prevents the establishment of an apical domain in inside cells. This allows Amot and Lats to interact with AJs, probably through Nf2, and become activated. Yap and Taz are retained in the cytoplasm and Tead4 remains switched off. In the absence of Tead4 activity, the cells continue to express ICM markers and take on an ICM fate. In this way, the separation of two distinct cell populations that differ in both position and polarity allows for differential gene regulation and the specification of two different cell fates.

### (c) Segregation of the inner cell mass into embryonic epiblast and primitive endoderm

**Figure 3.**
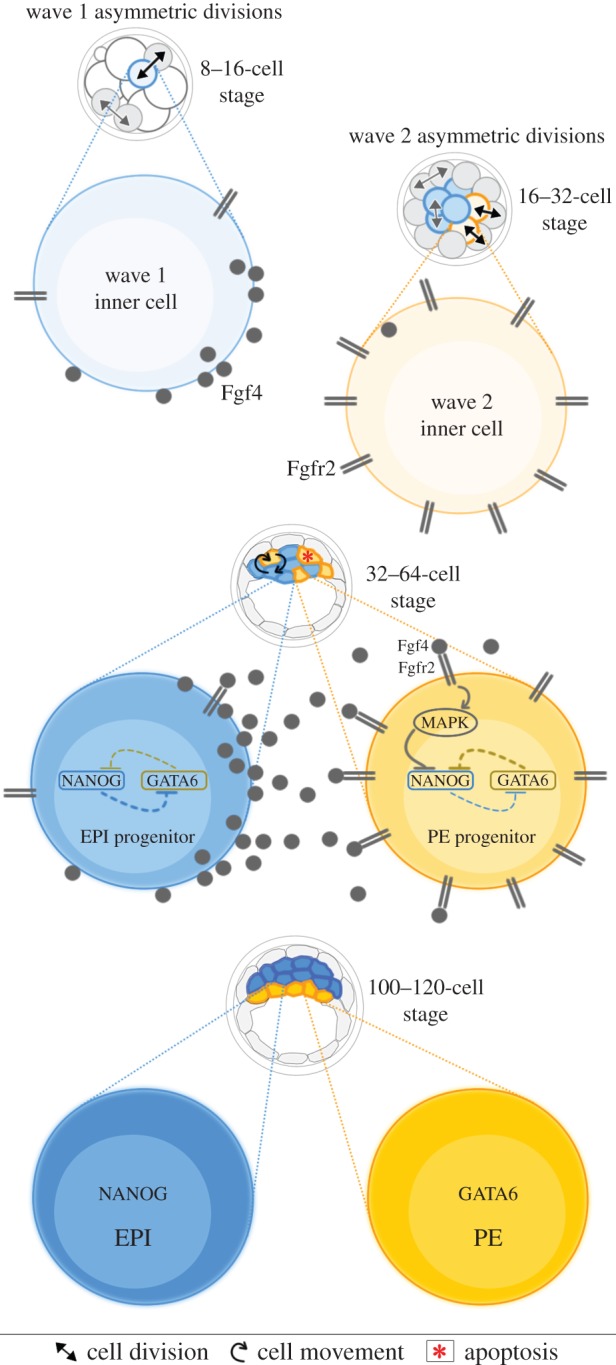
Specification of the EPI and PE. ICM cells internalized in the first wave of asymmetric cell divisions upregulate Fgf4, while cells internalized in the second wave express higher levels of Fgfr2, possibly by inheriting this transcription factor from their outside 16-cell stage mother cells. Fgf signalling in wave two cells inhibits the repression of Gata6 expression by Nanog, biasing these cells towards the PE lineage.

Traditionally, the segregation of the TE versus the ICM and the PE versus the EPI have been regarded as two separate cell fate decisions, however it is becoming clear that they are not independent. Precursors of the PE and EPI can be identified in the ICM by E3.5 when they start to activate unique transcriptional programmes [13,70]. EPI cells express pluripotency genes such as Nanog [38,71] and Sox2 [39], while PE cells activate an endodermal transcriptional network characterized by the expression of Gata6 [72], Gata4 [72], platelet-derived growth factor receptor alpha [15], Sox17 [73] and Sox7 [74]. The precursor cells are then sorted into the correct position for each lineage by a combination of active cell migration, positional induction and programmed cell death of incorrectly positioned cells [14,15]. By E4.5, the EPI occupies the deeper ICM compartment and PE cells line the surface of the ICM next to the blastocyst cavity. *Nanog* is the first gene to be specifically expressed in EPI cells and without its expression, the EPI fails to develop [75]. These *Nanog*^−/−^ embryos were also found to lack PE, suggesting that a signal provided by EPI precursor cells is critical to promote PE development. Fgf signalling has been uncovered as essential for PE fate specification [32,34]. EPI precursor cells in the early ICM upregulate expression of Fgf4, which is important for maintaining the PE fate programme in PE precursor cells, most likely by inhibiting the repression of Gata6 expression by Nanog [32,76]. As well as Nanog, Oct4, expressed throughout the ICM, is required for the expression of Fgf4 in EPI precursor cells and also for a newly discovered cell-autonomous role in the activation of PE gene expression [77]. The levels of Fgf signalling within the ICM are therefore critical for creating the correct balance of EPI and PE precursor cells (figure 3).

The generation of a mature ICM containing the appropriate number of EPI and PE cells is crucial for subsequent development. It has been recently demonstrated that despite the regulative ability of the preimplantation embryo, a minimum of four EPI cells has to be established by E4.5 for development to successfully progress beyond implantation [36]. Thus, the plasticity of ICM cells provides back-up mechanisms for maintaining this critical balance of ICM cell fate specification. The number of inside cells generated by each wave of asymmetric cell divisions is regulated such that each embryo will end up with roughly the same number of ICM cells: those with few cells internalized in the first wave will have more internalized in the second wave, and vice versa [2]. As cells internalized early switch on Fgf4, while those internalized later inherit higher levels of Fgfr2 from their outside parents [33,35], the amounts of Fgf ligand and receptor in the ICM is therefore modulated by the specific number of ICM cells generated in each wave. In embryos with few wave-one-derived inside cells, the levels of Fgf signalling are low resulting in only those wave-two-generated cells expressing high Fgfr2 forming PE. In embryos with many wave-one-derived inside cells, there is a high level of Fgf4 in the ICM which promotes the PE development of some wave-one-derived cells with lower levels of Fgfr2. In this way, the balance of EPI and PE remains the same regardless of the initial ICM composition. Interestingly, EPI precursors seem to have a more restricted cell fate potential than PE precursors [78]. It has been suggested that ICM cells internalized later in development inherit a greater flexibility and an increased ability to respond to differentiation cues than those internalized earlier due to exposure to the TE fate programme in outside cells [35]. In agreement with this flexibility, PE cells have been shown to not only contribute to extraembryonic lineages but also to some embryonic tissues after implantation [79]. These findings demonstrate the close relationship between the first two cell fate decisions and how the embryo is prepared for implantation through the concerted effects of gene expression, cell position, cell polarity and signalling on a highly flexible cell population.

## 3. Pre- to postimplantation transition: from blastocyst to egg cylinder

### (a) Blastocyst implantation

**Figure 4.**
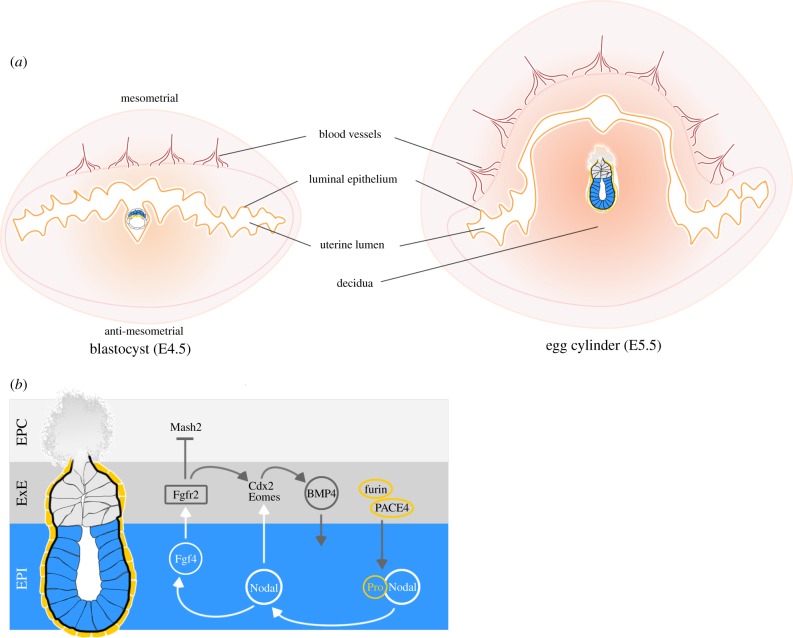
Implantation and signalling during egg cylinder formation. (*a*) After hatching, blastocyst adheres to the LE and invades the stroma at the antimesometrial site of the uterus. In response, the stromal cells differentiate into decidual cells that regulate trophoblast invasion, enable nutrients and gas exchange and ensure fetomaternal immune tolerance. In the next 24 h, the decidua rapidly proliferates to support embryo development into the egg cylinder and beyond. (*b*) The EPI cells secrete Fgf4 ligand that binds to Fgfr2 in the ExE in a paracrine manner. Active Fgfr2 signalling promotes Cdx2 and Eomes expression and inhibits genes associated with TS cell differentiation such as Mash2. Nodal produced in the EPI promotes ExE maintenance directly by activating core TS cell genes and indirectly by sustaining Fgf4 expression. In turn, ExE potentiates Nodal activity by secreting furin and PACE4 proteases that cleave out the Nodal propeptide to generate a mature ligand with higher activity.

When all three preimplantation cell lineages have been segregated, the blastocyst enters the uterus and hatches out of the zona pellucida. This process is a natural selection checkpoint as it permits the developmental progress only of embryos that are able to hatch. Within the next hours, the hatched blastocyst invades the maternal tissues and implants. Ovarian oestrogen and progesterone synchronize the timing of uterine receptivity with embryo development to ensure successful implantation and the level of oestrogen determines the duration of the implantation window within which the implantation process can occur [80]. The process of implantation can be divided into three phases: apposition, attachment and penetration. During the apposition phase, the diameter of the uterine lumen becomes reduced to position the floating embryo close to the luminal epithelium (LE). The first contact between the embryo and the mother is mediated by interdigitation of TE and LE microvilli, but this is insufficient for stable attachment. An anti-adhesive glycoprotein layer of mucins covers the uterine surface and serves as a barrier against pathogens. At the time of uterine receptivity, this layer is removed under the control of maternal hormones and actively by the embryo [81,82]. Only then does the combined effect of a variety of adhesion systems such as integrins and cadherins at the interface between the TE and LE mediate stable attachment of the embryo [83,84]. This direct contact induces apoptosis of LE at the site of attachment, allowing penetration of the TE into the endometrial stroma [85,86]. The embryo invades the antimesometrial site of the uterus with the ICM oriented towards the mesometrial pole (figure 4*a*). The surrounding stromal cells proliferate and differentiate into polyploid decidual cells [87] and the decidua supports the development of the embryo by enabling nutrients and gas exchange, ensuring fetomaternal immune tolerance by restricting the entry of cells of the immune system and regulating TE invasion [88,89].

In mouse, the mural TE (that surrounds the blastocyst cavity) makes the first contact with maternal tissues and subsequently differentiates into primary TE giant cells (TGCs). This is the first terminally differentiated cell type generated during prenatal development. Leading implantation, TGCs invade the uterine stroma and secrete factors such as progesterone and type I interferon that promote decidual cell differentiation [90,91]. Local vasculature remodelling and angiogenesis are induced at the implantation site by TGC subtypes to mediate nutrient, waste and gas exchange between the growing embryo and the mother [92]. In contrast to the terminal differentiation fate of the mural TE cells, the polar TE, which surrounds the outer surface of the ICM, is the source of multipotent progenitors—the TE stem (TS) cells. Following implantation, the polar TE proliferates and differentiates into the extraembryonic ectoderm (ExE) and ectoplacental cone (EPC) that build the proximal half of the egg cylinder, and later the placenta. TE differentiation depends on the T-box transcription factor Eomes, which functions downstream of Cdx2 in the TE fate programme. Accordingly, Eomes inactivation results in failure of the TE differentiation programme and developmental arrest at E4.5 [40,42]. After implantation, a self-renewing TS cell population is maintained in the ExE compartment that provides progenitors to the EPC. The maintenance of this stem cell pool in the ExE depends on expression of Elf5 that is in a positive feedback loop with Cdx2 and Eomes. In the absence of Elf5, Cdx2 and Eomes are shut down, leading to rapid depletion of the multipotent TE cells and loss of ExE by E5.5 [93,94]. Ets2 promotes the Elf5/Cdx2/Eomes expression circuit within the ExE. Accordingly, Ets2 knockout embryos implant but show severely reduced development of the TE lineages [95,96].

### (b) Initiation of proliferation following implantation

While during preimplantation development cleavage divisions generate progressively smaller blastomeres with constant total volume, following implantation a burst of cell proliferation initiates growth. This growth results in the expansion of embryonic and extraembryonic lineages into the blastocyst cavity around E5.0. At the same time, motile parietal endoderm cells come to line the internal surface of the mural TE, sandwiching a basement membrane layer (Reichert's membrane). Visceral endoderm cells derived from the PE cover both the EPI and ExE compartments of the elongating egg cylinder. As a result of this growth and reorganization, the embryo adopts a new shape within just 24 h following the initiation of implantation. Several signalling pathways have been uncovered as essential for the growth and survival of the embryo following implantation. The mammalian target of rapamycin (mTOR) regulates cell growth by integrating upstream signals of growth factors and amino acids. Cell proliferation is severely affected in all lineages of mTOR knockout embryos and they fail to progress beyond E5.5 [97,98]. Growth factors such as Igf and insulin promote cell growth by activating mTOR, through the PI3K/Akt pathway [99]. Genetic ablation of the p110β regulatory subunit, essential for the activity of the class IA PI3K, results in lethality during late pre- and early post-implantation stages [100]. Severe growth retardation and substantially reduced mTOR activity are observed in class 3 PI3K (PI3KC3) knockout embryos that die shortly after implantation [101]. *In vitro*, embryonic stem (ES) cell-specific Ras-like protein ERas activates PI3K to sustain ES cell proliferation. Upon xenotransplantation, ERas functions as an oncogene essential for the tumourigenic potential of ES cells [102]. Targeted disruptions of an array of genes associated with cancer in adult tissues result in early embryonic lethality, indicating an importance of genome integrity and cell survival during early embryogenesis. For example, loss of *Brca1* and *Brca2*, associated with breast and ovarian cancers, leads to death at early postimplantation stages due to inhibited cell proliferation [103–107].

Egg cylinder growth, organization and primitive streak formation to initiate gastrulation require ActRIB (activin type I), ActRIIA and ActRIIB (activin type II) receptor signalling [108,109]. Several TGFβ family ligands, such as Activin, Nodal and the mammalian Vg1 homologues GDF1 and 3, can bind these receptors. The activated receptors phosphorylate Smad2/3 that complex with Smad4 and translocate into the nucleus to activate target gene expression [110,111]. Smad2/3 and 4 are ubiquitously expressed and function as tumour suppressors in adult tissues [112]. The disruption of Smad2 or Smad4 causes defects in egg cylinder elongation and mesoderm induction [113,114]. Bone morphogenetic protein (BMP) ligands, such as BMP4, activate another branch of TGFβ signalling through the Smad1/5/8 pathway. BMP4 signalling is critical for EPI proliferation, mesoderm formation and induction of primordial germ cell fate [115,116]. The rapid proliferation and organization of embryonic and extraembryonic tissues following implantation is therefore regulated by the coordinated activity of multiple signalling pathways and extensive crosstalk between different cell types.

### (c) Development of the trophoblast

TS cells can be derived in culture from polar TE or ExE explants up to E8.5 [117,118]. TS cell proliferation and self-renewal *in vitro* requires the presence of Fgf4 and embryonic fibroblast conditioned medium [117]. Activin or TGFβ can replace the conditioned medium, although these factors are dispensable for the maintenance of the TS cell population in the embryo [119]. *In vivo* Fgf4 and Nodal signalling crosstalk between the ExE and the EPI enables the synchronous development of the egg cylinder (figure 4*b*). EPI cells produce Fgf4 that binds to Fgfr2 on the surface of the TE cells; loss of key components of the Fgf signalling pathway results in peri-implantation lethality due to failures not only in PE specification but also in TE maintenance [13,120–123]. Fgfr2 signalling controls TE cell survival through the downstream Shp2 phosphatase that triggers the Sfk/Ras/Erk signalling cascade. Erk1/2 kinases phosphorylate and target for degradation the pro-apoptotic protein Bim and a failure to activate Erk in Shp2 knockout embryos results in peri-implantation lethality and TS cell death *in vitro* even in the presence of Fgf4 [124]. Expression of genes essential for the maintenance of multipotent TE progenitors is also dependent on Fgf signalling. For example, the membrane-linked docking protein Frs2α is phosphorylated in response to Fgf4 stimulation of Fgfr2 that activates the downstream Erk cascade and promotes Cdx2 expression. Cdx2 in turn binds to the responsive enhancer element of the *Bmp4* promoter and upregulates Bmp4 expression [125,126]. Bmp4 produced in the ExE has been reported to act as a paracrine factor essential for proper EPI development after implantation [116]. Thus, Fgf4 signals transmitted between the distal and proximal parts of the egg cylinder cross-regulate directly and indirectly the maintenance and differentiation of the embryonic and extraembryonic lineages.

Fgf4 expression in the EPI is maintained by Nodal, a member of the TGFβ ligand superfamily that binds to type I and type II receptor dimers that, in turn, activate the downstream Smad2/Smad3 signalling cascade [111]. Nodal is secreted from EPI cells as a propeptide that is proteolytically processed extracellularly by proteases secreted by the ExE: furin (SPC1) and PACE4 (SPC4) [127]. Thus, while maturation of Nodal is under ExE control, Nodal signalling promotes ExE maintenance indirectly by sustaining Fgf4 expression in the EPI that, in turn, activates Fgfr2 signalling in the ExE. This results in the sustained expression of TS cell markers such as Cdx2, Eomes and Err2 and suppression of differentiation markers such as the paternally imprinted gene *Mash2*. Nodal also acts directly on the ExE in a paracrine manner, alongside Fgf4, to maintain the self-renewing population of TS cells. Accordingly, loss of Nodal or its convertases furin and PACE4 drives differentiation of the ExE towards an EPC fate [128].

The stem cell pool of the ExE contributes to the EPC that later gives rise to the spongiotrophoblast and secondary TGCs [129,130]. The basic helix-loop-helix (bHLH) transcription factor Mash2 regulates TGC differentiation in the EPC [131–133]. In TS cells, Mash2 is upregulated by nuclear Sp1 that binds a consensus Sp1 binding motif in the *Mash2* promoter. Activation of the PI3K/Akt pathway by TSSC3 leads to Sp1 nuclear translocation and Mash2 upregulation [134]. TGC differentiation requires Mash2 silencing by the polycomb group protein Eed and therefore ablation of Eed leads to permanent expression of Mash2 in the EPC and, consequently, reduced differentiation into secondary TGCs [135]. Low numbers of TGCs are also found in embryos deficient for the bHLH repressor I-mfa, proposed to inhibit Mash2 by preventing nuclear import and DNA binding [136]. Other bHLH transcription factors, such as Hand1 and Stra13, can override the inhibitory effects of Fgf4 signalling in TS cells and directly promote TGC differentiation [137].

### (d) Epiblast morphogenesis

A global reorganization of the EPI during the peri-implantation stages reshapes it from a compact ball of non-polarized cells into a cup-shaped polarized epithelium surrounding the pro-amniotic cavity. The emergence of this new organization of the pluripotent EPI provides the foundation for patterning and specification of the germ lineages that build the embryo proper. There are two major paths that can be followed to establish luminal space: hollowing and cavitation. In the process of cavitation, programmed cell death eliminates the inner cells of a solid cohort, generating an empty space. Hollowing, in contrast, does not require apoptosis but organized radial polarization and separation of apical membranes to form a central lumen [138,139]. Embryoid bodies (EBs) have been commonly used as an accessible *in vitro* model that recapitulates many aspects of embryonic development. Studies of aggregates of ES cells or embryonic carcinoma cells grown in suspension indicated that apoptosis of the cells in the core of EBs is required for cavity formation [140] with BMP and Rac1 signalling proposed to promote the elimination of inside cells and the survival of cells contacting the basement membrane, respectively [141,142]. Although EBs are a valuable *in vitro* model, they lack proper embryonic organization and therefore may not be able to recapitulate the physiological processes that occur *in vivo*. For example, EBs and embryos differ in their initial cell number. While EBs contain a few hundreds of cells on the first day of culture, the EPI of the implanting blastocyst consists of only 8–16 cells [36,143]. The second difference is timing. While cavitation and the establishment of polarized epithelium in EBs is a slow process that takes several days, the blastocyst to egg cylinder transition occurs *in vivo* within a 24 h period beginning at E4.5 [144]. Thus, the large number of slowly polarizing cells in the EBs may induce apoptotic-mediated cavitation, as indeed is observed in high-density culture of MDCK cells in the absence of strong polarization cues. By contrast, low-density MDCK cells efficiently polarize and form lumens within 2 days through a process of hollowing that does not require cell death. Therefore, cells can use different mechanisms for lumen formation depending on the polarization efficiency [145].

**Figure 5.**
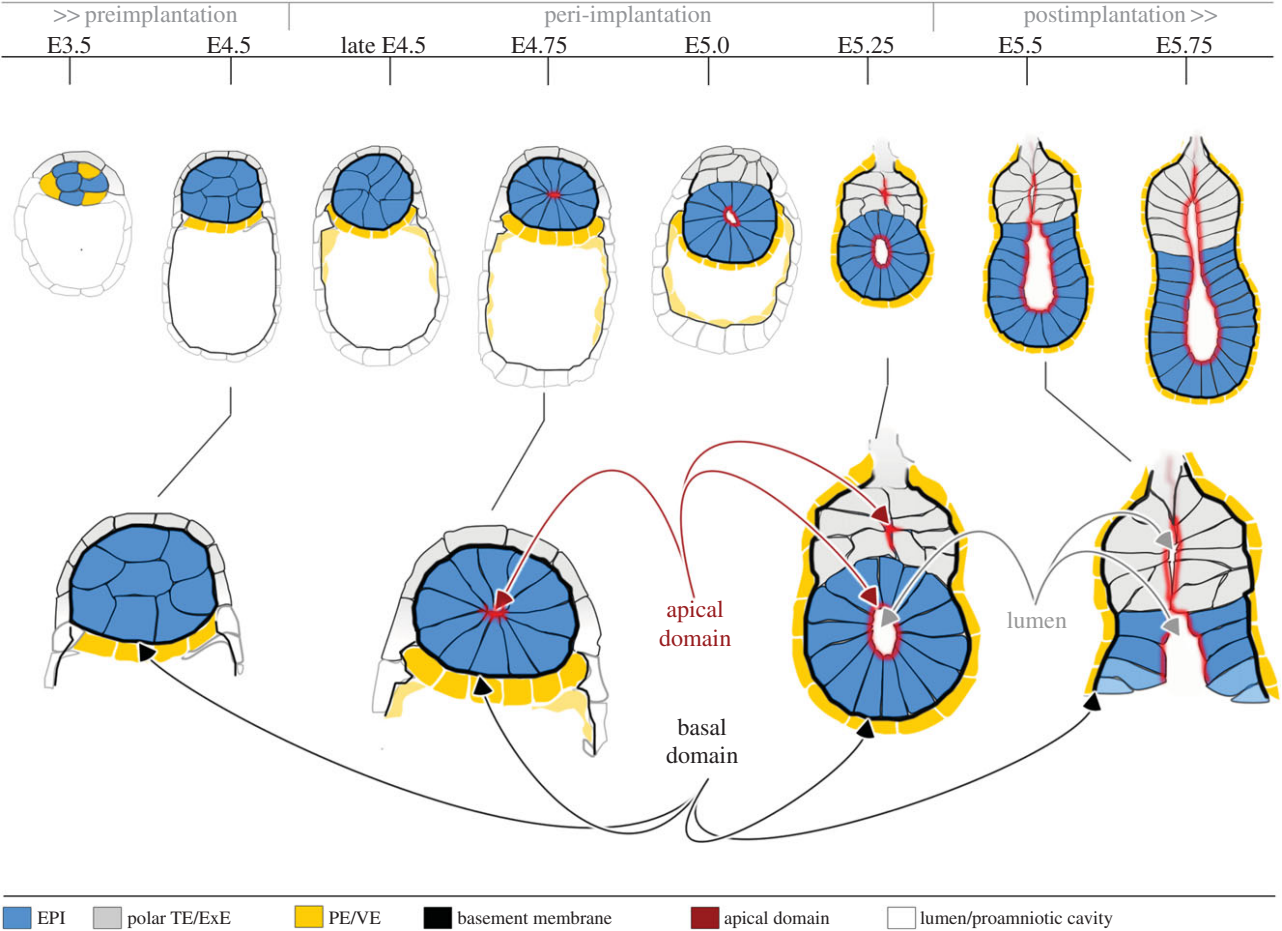
Model of peri-implantation morphogenesis. Following preimplantation lineage segregation (E3.5–E4.5), the extraembryonic lineages start secreting ECM proteins that assemble a basal membrane that wraps around the EPI and provides polarization cues through integrin receptors. During the peri-implantation period (late E4.5–E5.0), the pluripotent EPI cells establish apical–basal polarity, change shape and constrict apically while clustering to form a rosette. A central lumen emerges in the centre of the rosette through hollowing of apical membranes by charge repulsion (E5.0–E5.25). As the egg cylinder elongates the lumen enlarges and incorporates intramembranous spaces of the proximal ExE to form the mature pro-amniotic cavity (E5.5–E5.75).

With an aim to understand how the EPI is reorganized during peri-implantation stages, we have established an *in vitro* environment that supports development from the preimplantation blastocyst to the postimplantation egg cylinder stage [146]. This has allowed us to reveal the morphogenetic steps taken by the embryo at its pre- to post-implantation transition [147]. Using a cell death reporter and genetic and pharmacological approaches to inhibit cell death, we have found that apoptosis is not required for EPI morphogenesis and pro-amniotic cavity generation. Moreover, there are no signs of cell death in the region where the cavity would form in either peri-implantation embryos cultured *in vitro* or recovered from mothers. To reveal any alternative mechanism for cavity formation, we followed the organization of EPI cells at the time of pre- to post-implantation transition [147]. This revealed that the EPI becomes reorganized from a ball of unpolarized cells into a highly organized rosette-like structure at the time of implantation, a process involving drastic changes in cell shape and polarization (figure 5). This reorganization appears to be a result of apical constriction mediated by contraction of the actomyosin network linked to AJs. As cells acquire a polarized epithelial morphology, actin filaments accumulate apically and the Golgi apparatus and nucleus localize sub-apically and basally, respectively. As development progresses, a single lumen emerges in the centre of the rosette [140]. Lumen formation is likely to be a result of membrane separation through charge repulsion as the apical domains facing the lumen express the highly negatively charged sialomucin podocalyxin (PCX) involved in lumen formation in glomerular and MDCK cells [148,149]. Thus, formation of a polarized rosette is the first morphogenetic step that reshapes the EPI during implantation and serves to provide a foundation for the emerging egg cylinder.

But what triggers the self-organization of EPI cells into a highly polarized rosette? Our results indicate that this is mediated by extracellular matrix (ECM) signalling through integrin receptors to direct cell polarization [147]. ECM proteins, such as laminin secreted by the PE and TE, assemble a basement membrane that envelopes the EPI of the implanting blastocyst. The function of this basement membrane can be mimicked *in vitro* by embedding ICMs in matrigel, leading to the formation of polarized EPI-like structures with a central lumen. The basement membrane therefore could be seen as creating a niche that provides polarization cues to the maturing EPI [147]. This is consistent with laminin and integrin functions in the early embryo as elimination of the laminin-γ1 subunit leads to a failure to assemble the basement membrane, resulting in peri-implantation lethality [150], and elimination of β1-integrin receptor leads to EPI defect following implantation [151,152]. Strikingly, it appears that the process of EPI morphogenesis can be mimicked *in vitro* by embedding small clumps of ES cells into three-dimensional ECM gels. The ECM proteins trigger polarization and lumenogenesis through β1-integrin receptors and, as in the peri-implantation EPI, the cells change shape and constrict apically within the centre of the ES cell sphere. A single PCX-coated lumen emerges in the centre of the radially arranged cells, resembling the morphogenesis following implantation [147].

Overall, the discovery of the self-organizing properties of pluripotent EPI cells leads to a new model of the morphogenetic steps of the blastocyst to egg cylinder transition (figure 5). It proposes that prior to implantation (E4.5), the extraembryonic lineages of the late blastocyst consist of polarized epithelial cells that secrete ECM proteins, which assemble a basement membrane that wraps around the EPI. This basement membrane creates a niche where ECM components are sensed by integrin receptors on the surface of the EPI. The niche provides polarization cues that orient the apical–basal axis of the EPI cells. Actomyosin constriction and accumulation of apical determinants of the Par complex reorganize the EPI into a radially polarized rosette-like structure (E4.75–E5.0). Among other proteins, anti-adhesive molecules such as PCX are delivered to the apical surfaces in the centre of the rosette and, as a result, membrane repulsion leads to hollowing and a central lumen emerges. A similar process of polarization and hollowing is likely to occur in the ExE with both lumens then merging to form the mature pro-amniotic cavity (E5.5–E5.75). The lumen enlarges as the egg cylinder elongates and active processes of exocytosis and pumping can also potentiate accumulation of fluid leading to pro-amniotic cavity expansion. As the egg cylinder enlarges, the basement membrane separating the EPI and the ExE is no longer maintained. Thus, the basement membrane resembles a basket, structurally linked to the EPI cells through integrin-mediated contacts, acting as a mould to direct the shape of the EPI, transforming it from a symmetric hollowed sphere into a cup, building the distal part of the mature egg cylinder.

### (e) Concluding remarks

Implantation is a unique property of mammalian embryo development and the morphogenetic processes driving the pre- to postimplantation transition have only recently begun to be understood. As imaging of developmental dynamics and individual cell-tracking techniques improve, so does our understanding of how the early embryo prepares the necessary cell types for implantation and then transforms itself to initiate development of the embryo proper.
